# The influence of moral reasoning on adolescent decision-making and stress responses during VR social-moral conflict

**DOI:** 10.3389/fpsyg.2025.1661490

**Published:** 2025-10-15

**Authors:** Jianbao Zhang, Tingting Zhang

**Affiliations:** ^1^School of Marxism, Beijing Wuzi University, Beijing, China; ^2^Student Affairs Department, Huaiyin Normal College, Huai'an, China

**Keywords:** moral reasoning, adolescent stress, social-moral conflict, virtual reality (VR), heart rate, decision-making, state anxiety, buffering effect

## Abstract

**Introduction:**

Moral reasoning is a key component of moral development, yet its role in regulating stress during high-pressure social conflicts in adolescents is underexplored. This study investigated the influence of moral reasoning ability on decision-making and stress responses in Chinese adolescents during a simulated social-moral conflict.

**Methods:**

Chinese adolescents (*N* = 118, 14–17 years) were grouped into high and low moral reasoning ability based on their Defining Issues Test-2 (DIT-2) scores. In a counterbalanced design, they were exposed to a high-pressure social-moral conflict scenario in immersive virtual reality (VR) and a neutral control condition. We measured physiological stress (heart rate), psychological stress (state anxiety), and moral decision-making (accuracy, speed).

**Results:**

The VR scenario successfully induced greater physiological arousal (higher heart rate) and state anxiety compared to the control condition. However, the level of moral reasoning ability had no significant effect on moral decision-making accuracy or state anxiety. A non-significant trend suggested that high-reasoning participants exhibited slightly lower average heart rates during the VR conflict, hinting at a potential stress-buffering effect.

**Discussion:**

The findings indicate that the practical application of moral reasoning in adolescents is strongly moderated by situational pressures and developmental factors. While higher moral reasoning may not buffer against acute stress or improve immediate decision-making in intense social conflicts, this study highlights the value of VR for examining moral behavior in ecologically valid contexts. Future research with larger samples is needed to verify the potential stress-buffering trend.

## 1 Introduction

Moral Intelligence (MI) is a key construct for understanding the cognitive and emotional processes behind ethical behavior (Huy and Phuc, 2024; Tanner and Christen, 2014). It is defined as the ability to discern right from wrong and act on ethical convictions, encompassing components like moral identity, empathy, and moral reasoning (Lennick and Kiel, 2011; Narvaez, 2010). This study focuses specifically on moral reasoning: the cognitive process of determining an ethical course of action based on principles and outcomes (Bucciarelli et al., 2008). Rooted in Kohlberg's (1984) stage theory, moral reasoning is thought to progress from self-centered logic to the application of universal principles, a crucial skill for navigating complex social dilemmas.

Despite extensive research on the broader MI construct, the specific role of moral reasoning in regulating stress during high-pressure social conflicts remains underexplored, particularly in adolescents. Adolescence is a critical period of cognitive and emotional growth, marked by heightened social sensitivity and the formation of a moral identity (Kohlberg, 1984; Snarey et al., 1985). While studies link MI to better ethical decisions (Tanner and Christen, 2014), its function during acute, socially complex stress is less understood.

Research suggests that well-developed MI may support stress management (Asefi Far et al., 2023), yet the immediate pressure of real-world moral dilemmas can overwhelm the cognitive resources of adolescents, whose regulatory systems are still maturing (Steinberg, 2005). To investigate this gap, the present study uses an immersive Virtual Reality (VR) environment to simulate a realistic social-moral conflict. VR offers a methodologically rigorous platform to examine how moral reasoning ability influences stress responses and decision-making, providing a high degree of experimental control and ecological validity (Ni et al., 2023; Parsons and Rizzo, 2008).

Based on this theoretical framework, we tested the following specific hypotheses:

1. The VR social-moral conflict scenario would successfully induce stress, resulting in significantly higher physiological arousal (heart rate) and self-reported state anxiety compared to a neutral control condition.

2. Moral reasoning ability would serve a stress-buffering function. We predicted that adolescents with higher moral reasoning ability would exhibit attenuated physiological and psychological stress responses during the VR conflict compared to those with lower moral reasoning ability.

3. Higher moral reasoning ability would be associated with more effective decision-making under pressure. We predicted that participants in the high-reasoning group would demonstrate greater decision-making accuracy within the VR scenario.

## 2 Literature review

### 2.1 Theoretical background

Moral intelligence (MI) involves the cognitive and emotional processes underlying ethical behavior and decision-making (Kornilova and Chigrinova, 2014), defined as the ability to distinguish right from wrong, uphold ethical convictions, and act accordingly despite challenges (Lennick and Kiel, 2011). As a multifaceted construct, MI integrates key components such as moral reasoning, empathy, and moral identity to shape moral actions.

Moral reasoning, a core MI component, entails evaluating ethical rightness or wrongness based on principles and outcomes (Rest et al., 1999). Rooted in Kohlberg's (1984) stages of moral development, it evolves from self-focused logic to principled thinking, with advanced reasoning linked to higher MI and better navigation of complex dilemmas (Gibbs et al., 2007). Empathy, another key element, encompasses understanding (cognitive empathy) and sharing (affective empathy) others' emotions (Davis, 1983). It fosters compassion and prosocial behavior, with research showing higher empathy correlates with ethical adherence and helping actions (Eisenberg et al., 2006; Krol and Bartz, 2022). Together, moral reasoning and empathy build moral identity—the degree to which being moral is central to one's self-concept (Aquino and Reed, 2002; Boegershausen et al., 2015)—enhancing consistency between beliefs and actions (Hannah et al., 2020; Hardy and Carlo, 2005).

Social-moral conflict, where personal values clash with social norms (Lehalle, 2020; Turiel, 2002), also shapes MI and decision-making (Weber, 2019). Such conflict can cause cognitive dissonance and distress, especially under pressure or when observing unethical acts (Chowdhury, 2017; Rest et al., 1999). Its effects vary: it may foster moral growth by reinforcing values (Blasi, 1980; Chorus, 2015) or lead to moral disengagement if unresolved, prompting rationalized unethical behavior (Bandura, 1999; White et al., 2009). Effectively managing social-moral conflict is thus a critical MI skill, emphasizing the need to develop moral reasoning and empathy across contexts (Bajovic and Rizzo, 2021).

### 2.2 Moral intelligence and decision-making

Moral intelligence (MI) plays a significant role in decision-making processes, particularly during adolescence, a key period of cognitive and emotional growth. Research exploring the link between MI and ethical decision-making reveals complexity. While higher MI levels generally correspond with more ethical choices and enhanced decision-making capabilities under pressure and uncertainty, inconsistencies in the findings necessitate a closer examination of this relationship.

Substantial research indicates that higher MI promotes more ethical decision-making. Adolescents with greater MI demonstrate stronger abilities to discern right from wrong, align decisions with ethical principles, and resist unethical temptations (Lennick and Kiel, 2011; Tanner and Christen, 2014). Tanner and Christen's (2014) framework, for example, shows high-MI adolescents are more prone to engaging in prosocial behaviors and avoiding actions that could harm others. This association is further supported by studies on MI's core components, including moral reasoning and empathy, finding that strength in these areas correlates with more ethical decisions (Rest et al., 1999; Small and Lew, 2021).

MI's influence on decision-making appears particularly pronounced under conditions of pressure or uncertainty. Such conditions often demand rapid moral judgments, making well-developed moral competencies essential for adolescents. For instance, higher MI facilitates balancing ethical considerations with practical outcomes in sustainable decision-making during uncertain times (Huy and Phuc, 2024). Relatedly, emotional intelligence, which is closely linked to MI, helps individuals manage ethical dilemmas in high-stress environments, suggesting that higher MI fosters resilience and adherence to ethical standards under pressure (Holian, 2006).

Despite these positive findings, the literature reveals inconsistencies regarding MI's direct impact on ethical decisions. Some studies do not find unequivocal support, suggesting other factors are at play. For example, personal values can sometimes overshadow MI's influence on choices in interpersonal interactions (Kornilova and Chigrinova, 2014). Moreover, a systematic review highlights that the influence of MI on high-stakes moral decisions varies significantly across different contexts and cultures (Ni et al., 2023). This variability suggests that situational pressures, a key focus of the present study, may be a critical and underexplored moderator in the relationship between moral reasoning and behavior.

### 2.3 Moral intelligence and stress regulation

Moral Intelligence (MI) is increasingly recognized for its role in ethical decision-making and effective stress management, particularly in challenging social contexts (Barida et al., 2019; Lennick and Kiel, 2011). This connection stems from MI's core components—principally moral reasoning and empathy—which provide the cognitive and emotional resources for navigating stressful situations (Asefi Far et al., 2023).

The cognitive faculty of moral reasoning, which involves assessing ethical principles to guide behavior (Rest et al., 1999), is central to this process. By enabling individuals to maintain ethical clarity under pressure, strong moral reasoning reduces cognitive dissonance and grounds their actions in a stable moral framework, thereby alleviating stress (Bucciarelli et al., 2008; Gibbs et al., 2007). Supporting this, (Asefi Far et al. 2023) found that MI, particularly its moral reasoning component, was inversely linked to student anxiety, an effect mediated by distress tolerance. Complementing this is empathy—the capacity to understand and share others' emotions (Davis, 1983)—which mitigates stress by fostering supportive social interactions. For instance, studies on emotional intelligence (a related construct inclusive of empathy) have associated it with lower moral distress in nurses and improved stress management in adolescents (Awad and Ashour, 2020; Pérez-Fuentes et al., 2019).

Although direct research on MI and stress regulation is still developing, insights from studies on Emotional Intelligence (EI) are highly relevant due to the constructs' significant overlap. Adolescents with higher EI demonstrate more effective coping strategies and resilience under pressure (Lea et al., 2023), suggesting MI supports similar capabilities. However, the link is not absolute. The effectiveness of MI in buffering stress can be moderated by external factors, as the influence of emotional competencies varies by population (Irfan and Kausar, 2018) and can be shaped by family dynamics and parenting styles (Alhadabi et al., 2019). These findings underscore that while MI is a valuable internal resource for managing stress, its impact is often contingent on the surrounding social context.

### 2.4 The role of social-moral conflict

Social-moral conflict arises when personal moral values clash with social expectations, norms, or pressures (Snarey, 1985; Wallace, 2019). This conflict is particularly significant during adolescence, a period of heightened social sensitivity and ongoing moral identity formation (Buon et al., 2016). It strongly affects stress levels and decision-making, requiring adolescents to navigate complex ethical situations and reconcile competing values (Smetana, 2011; Turiel, 2002; Wallace, 2019).

Social-moral conflict typically pits an individual's moral compass against external pressures (Blasi, 1980; Turiel, 2002), such as peer pressure toward unethical acts, cultural norms opposing personal values, or institutional policies challenging beliefs (Fourie, 2015). This tension can harm psychological wellbeing and impair decision-making by generating cognitive dissonance—mental discomfort from conflicting beliefs—that heightens stress and disrupts judgment (Blasi, 1980; Gibbs et al., 2007; Lehalle, 2020; Rest et al., 1999).

Adolescents are especially susceptible due to their developmental stage, balancing moral value consolidation with peer conformity pressures (Kohlberg, 1984; Snarey et al., 1985). This dual need—to form a moral identity and gain social acceptance—complicates navigating conflicting viewpoints, often causing significant stress and hindering decision-making (Cooley et al., 2012; Eisenberg et al., 2006). Moral distress in young professionals facing ethical conflicts further illustrates this stress-decision-making link (Awad and Ashour, 2020). Social pressure amplifies these effects in conformity-driven settings, pushing adolescents toward group norms over personal values, increasing stress and risking unethical choices (Buon et al., 2016; Turiel, 2002). Family and peer expectations also shape the development of moral intelligence (MI) and decision-making (Alhadabi et al., 2019). Yet, MI components—such as advanced moral reasoning and empathy—can lessen these negative impacts (Bajovic and Rizzo, 2021; Davis, 1983; Gibbs et al., 2007). Strong reasoning skills help adolescents analyze and resolve conflicts, reducing stress and supporting ethical decisions, while empathy aids stress regulation (Downey et al., 2010). However, social-moral conflict's effects are not solely adverse. It can foster moral growth and resilience by challenging adolescents to refine reasoning and reinforce convictions through diverse perspectives (Blasi, 1980; Ni et al., 2023; Turiel, 2002), highlighting its dual role in development.

### 2.5 Research gap and rationale for the current study

Although Moral Intelligence (MI), social-moral conflict, and decision-making are individually well-studied, their dynamic interplay during adolescence remains largely unexplored. Current literature typically examines these factors in isolation: the influence of MI on ethical choices (Rest et al., 1999; Tanner and Christen, 2014), the impact of conflict on stress (Blasi, 1980; Turiel, 2002), or performance under pressure (Huy and Phuc, 2024). This fragmented approach overlooks their combined effects, which is a significant omission given the heightened developmental vulnerability of adolescents' moral and cognitive faculties (Kohlberg, 1984). Furthermore, while social context is known to shape moral behavior (Alhadabi et al., 2019; Smetana, 2011), it remains unclear how an adolescent's moral reasoning ability—a key component of MI—interacts with situational social-moral conflict to shape decision-making under stress.

The present study is designed to address this critical gap by investigating whether moral reasoning ability moderates the relationship between social-moral conflict and adolescent decision-making under pressure. To create an ecologically valid context that simulates the social pressures of real-world dilemmas (Buon et al., 2016), we employ immersive Virtual Reality (VR), a proven method for inducing stress and modeling complex social interactions (Ni et al., 2023). Our central aim is to determine if higher moral reasoning ability acts as a protective buffer, mitigating the adverse effects of conflict on an adolescent's decisions and stress responses. By combining objective physiological and performance data (heart rate, decision-making tasks) with subjective stress reports, this study offers a multi-faceted analysis that moves beyond existing work to clarify the specific mechanisms through which moral intelligence may function in high-stakes, social-moral scenarios.

## 3 Method

### 3.1 Participants

Participants were 118 adolescent students (aged 14-17 years) recruited via convenience sampling from two senior high schools in Beijing, China. A multi-faceted recruitment strategy involved collaboration with school personnel, informative posters displayed on school bulletin boards, teacher announcements emphasizing voluntary participation, and online advertisements on relevant social media platforms (e.g., WeChat, Weibo). All recruitment materials clearly outlined the study's purpose, eligibility criteria, time commitment, and contact information, adhering to ethical guidelines.

Inclusion criteria required participants to be 14-17 years old, fluent in Mandarin Chinese, possess normal or corrected-to-normal vision, and have no history of diagnosed neurological or psychiatric conditions (confirmed via parental/self-report). Participants were excluded if they could not understand study procedures or provide informed assent, were currently enrolled in other psychological intervention studies, or were unable or unwilling to complete the virtual reality (VR) task due to potential motion sickness or claustrophobia.

Participant confidentiality and ethical treatment were prioritized throughout the study. Written informed consent was obtained from parents or legal guardians for all participants (under 18 years), detailing the study aims, procedures, potential risks and benefits, participant rights, and withdrawal options. Following parental consent, interested students attended an information session where procedures were explained in age-appropriate language. Students had the opportunity to ask questions before providing written assent, confirming their voluntary participation.

An a priori power analysis conducted using G^*^Power (Faul et al., 2007) for a mixed-factors ANOVA indicated a target sample size of 114 participants. This calculation was based on the goal of detecting a medium effect size (*f* = _0.25_) with 0.80 power at an alpha level of 0.05. Given the novelty of using an interactive VR paradigm for this research question, our choice of a medium effect size was guided by Cohen's (1988) conventions and a comprehensive meta-analysis on the influence of psychosocial factors on physiological stress reactivity (Chida and Hamer, 2008). This was determined to be the smallest effect size of practical and theoretical significance. To account for potential attrition, we aimed to recruit 120 participants. The final sample comprised 118 adolescents (60 female, 58 male) with a mean age of 15.8 years (*SD* = 0.72). Based on a median split of their Defining Issues Test-2 (DIT-2) N2-scores, participants were divided into high moral reasoning ability (*n* = 59) and low moral reasoning ability (*n* = 59) groups. The groups did not significantly differ in age, gender, or educational background, ensuring baseline comparability.

### 3.2 Measures

#### 3.2.1 Moral reasoning ability

Moral reasoning ability was assessed using the Defining Issues Test-2 (DIT-2; Rest et al., 1999), a well-established and widely used measure of moral development. For this study, we used the Chinese version of the DIT-2, which underwent a rigorous translation, linguistic review, and cultural validation process supervised by one of the test's original developers (Zhang and Thoma, 2016). This process was specifically designed to ensure that the moral dilemmas were comprehensible and that their conceptual meaning was equivalent for Chinese participants, thereby addressing potential cross-cultural subtexts.

This instrument presents participants with six short moral dilemmas and asks them to rank six different justifications for action based on their moral reasoning complexity. The DIT-2 N2-score, which ranges from 0 to 95, was used to assess moral reasoning ability. The N2-score is a composite index that reflects both the preference for post conventional moral reasoning and the ability to discriminate between higher and lower stage reasoning. The reliability of the DIT-2 is well-documented, with Cronbach's alpha typically exceeding 0.70, indicating acceptable internal consistency (Rest et al., 1999). Validity studies have shown that DIT-2 scores correlate significantly with measures of cognitive moral development and related constructs, supporting its construct validity.

#### 3.2.2 Stress induction

Social-moral conflict was induced using a novel, immersive virtual reality (VR) scenario developed in consultation with experts in moral development and conflict resolution. Participants were randomly assigned to lead a 20 m virtual classroom discussion featuring four diverse virtual classmates (avatars). The discussion focused on one of two pre-determined controversial social issues: the ethics of animal testing in medical research or the environmental impact of single-use plastics. These topics were selected for their relevance to adolescents, potential to evoke strong opinions, and capacity to elicit moral dilemmas, as confirmed through pilot testing and educator feedback. The virtual classmates engaged using pre-scripted responses representing both supportive and opposing viewpoints grounded in common real-world arguments on the issues (e.g., animal rights vs. potential medical benefits in the testing scenario).

To simulate escalating social pressure and induce stress, the intensity of the virtual classmates' arguments and disagreements gradually increased throughout the discussion. Participants also encountered unexpected interruptions and emotionally charged comments from the avatars, designed to challenge their ability to maintain composure and facilitate a productive dialogue effectively.

The scenario's effectiveness in inducing stress and social-moral conflict was validated in a pilot study with 10 adolescents (aged 14-17 years; 5 female, 5 male). Based on physiological data and post-scenario interviews from this pilot, we refined the final scenario by adjusting the timing of avatar interruptions and escalating the intensity of oppositional arguments in the final third of the discussion to maximize social pressure. This ensured the task was ethically sound and effective in eliciting the intended conflict. Table 1 provides a detailed breakdown and comparison of the components of the experimental and control conditions.

**Table 1:** Comparison of VR social-moral conflict and control conditions.

**Component**	**VR social-moral conflict condition**	**Control condition**
**Environment**	Immersive virtual classroom	Immersive virtual library
**Core task**	Moderate a 20 m controversial discussion	Organize virtual books by category for 20 m
**Social interaction**	Dynamic interaction with four emotionally expressive and increasingly confrontational avatars	None (solitary task)
**Moral content**	High; participants must navigate ethical dilemmas, value conflicts, and offensive remarks	None
**Primary stressor**	Escalating social pressure and interpersonal conflict	Minimal cognitive load from a simple sorting task
**Key variables**	Heart rate, state anxiety, decision accuracy, decision speed	Heart rate, state anxiety

#### 3.2.3 Physiological reactivity

Heart Rate (HR) was continuously measured throughout the experiment using a Biopac MP160 physiological recording system coupled with a finger plethysmograph. HR data were collected at a sampling rate of 1,000 Hz and preprocessed using artifact correction techniques to remove noise and ensure accurate readings. Baseline HR was measured during a 2 m resting period before the VR scenario. To assess physiological stress responses, the average heart rate during the VR social-moral conflict scenario and the control condition was compared. The HR during each condition was also compared against the individual's baseline HR, allowing for an understanding of condition-induced changes relative to a resting state. This method of measuring physiological reactivity is well-established and provides reliable indices of autonomic nervous system activity in response to stress (Berntson et al., 2007).

#### 3.2.4 Cognitive responses

**Decision-making accuracy (VR scenario only):** Throughout the immersive VR group discussions, participants encountered opportunities to make moral choices (e.g., addressing offensive remarks, deciding how to weigh differing viewpoints). The accuracy of these decisions was evaluated using a pre-determined rubric developed in consultation with moral psychology experts and grounded in Kohlberg's theory of post-conventional moral development, emphasizing principled reasoning based on justice, fairness, individual rights, and collective wellbeing. The rubric comprised five criteria, each scored on a 1 (low) to 5 (high) scale: (1) demonstrated respect for diverse viewpoints; (2) promotion of inclusive dialogue (e.g., encouraging open communication, active listening); (3) focus on finding common ground or shared values; (4) adherence to ethical principles (e.g., justice, fairness); and (5) overall effectiveness in facilitating a productive and respectful discussion. Two independent raters, trained in moral psychology and familiar with the VR scenario, coded participant responses. High inter-rater reliability was achieved (Cohen's κ > 0.80), supporting the consistency and validity of the coding process. This measure was applicable only to the VR social-moral conflict scenario, as the control condition did not involve moral dilemmas.

**Decision-making speed (VR SCENARIO ONLY):** The time taken for participants to make each moral decision within the VR scenario was recorded via the VR system's internal clock. This measure served as a proxy for cognitive processing speed under pressure, with shorter decision times interpreted as indicating more efficient processing (i.e., quicker evaluation of moral implications and selection of action). The validity of this measure was established in a separate pilot study where decision-making speed in the VR scenario significantly correlated with performance on standardized cognitive processing tasks (e.g., Stroop task, Flanker task) that assess cognitive control and processing speed. Similar to accuracy, this measure was specific to the moral dilemmas presented in the VR scenario.

#### 3.2.5 Emotional reactivity

Following the VR scenario, participants completed the State-Trait Anxiety Inventory-State Scale (STAI-S; Spielberger et al., 1971). The STAI-S is a self-report measure that assesses immediate anxiety levels in response to a specific situation. Participants rated 20 statements on a 4-point Likert scale, with higher scores indicating greater levels of state anxiety. The STAI-S is widely used in psychological research to measure situational stress and has demonstrated high reliability (Cronbach's alpha >0.90) and validity across diverse populations (Spielberger et al., 1971). The translated version used in this study maintains these psychometric properties, providing a robust measure of participants' immediate emotional reactivity following the stress-inducing VR scenario.

### 3.3 Procedure

The study was conducted in two separate sessions, spaced no more than one week apart, each meticulously planned to ensure participant comfort, understanding, and data integrity.

**Session 1: Informed Consent, Questionnaires, and Moral Reasoning Assessment (60 m)**

The initial session took place in a laboratory at participants' schools. A research assistant began by explaining the study's procedures and its voluntary nature, after which participants provided written informed consent or assent. Following this, participants completed a brief demographic questionnaire covering their age, gender, and educational background.

The main component of this session was the administration of the Defining Issues Test-2 (DIT-2) to assess moral reasoning ability. Upon completion, participants were classified into high or low moral reasoning groups based on a median split of their DIT-2 N2-scores. This method ensured a balanced distribution of reasoning abilities for the subsequent experimental phase of the study.

**Session 2: VR Scenario, Control Condition, Physiological Recording, and Debriefing (90 m)**

The second session took place in a dedicated VR laboratory, where participants were fitted with a high-resolution VR headset and a finger plethysmograph (Biopac MP160) for continuous Heart Rate (HR) measurement. To acclimate them to the virtual environment without altering their emotional state, the session began with a 5 m familiarization phase in a calming underwater scene. This was immediately followed by a 2 m resting baseline HR recording, establishing a physiological starting point before the main tasks.

With the baseline established, participants experienced both a 20 m social-moral conflict scenario and a 20 m control condition, presented in a counterbalanced order and separated by a 10 m rest period. The social-moral conflict scenario immersed participants in a virtual classroom discussion where four classmates' pre-programmed responses grew increasingly oppositional, designed to simulate social pressure. The control condition, by contrast, involved a non-social, non-moral task of organizing virtual books in a library, which was crafted to match the experimental scenario's duration and basic cognitive demands. Data collection was synchronized with these tasks; HR was recorded continuously throughout both conditions, and participants completed the State-Trait Anxiety Inventory-State Scale (STAI-S) to assess their immediate stress levels after each one. Assessments of decision-making accuracy and speed, however, were collected only during the social-moral conflict scenario. The entire session concluded with a semi-structured debriefing interview to gather qualitative data about participants' experiences, after which they were thanked and compensated.

### 3.4 Data analysis

All data analyses were performed using IBM SPSS Statistics version 27. Initial analyses included descriptive statistics (means, standard deviations, frequencies) for all study variables (demographics, moral reasoning ability scores (DIT-2 N2), average HR during each condition, moral decision-making accuracy and speed (for the VR scenario only), self-reported stress (STAI-S)) to summarize data characteristics and check assumptions for subsequent analyses (Field, 2018).

The primary analyses involved two separate 2 (Moral Reasoning Ability Group: High vs. Low) × 2 (Condition: VR Scenario vs. Control) mixed-factors ANOVAs to examine main and interaction effects. The first ANOVA assessed effects on average HR during the conditions to determine if moral reasoning ability moderated physiological stress responses to the social-moral conflict condition (Stevens, 2012). This analysis directly compared the average HR during the VR scenario and control tasks, examining the extent to which each condition elevated HR from a general resting state. The second ANOVA examined effects on moral decision-making accuracy (analyzed only for the VR scenario, with the control condition as a conceptual baseline for general cognitive demands), moral decision-making speed (analyzed only for the VR scenario, with the control condition as a conceptual baseline for general cognitive demands), and self-reported stress levels (STAI-S). Significant main or interaction effects from the ANOVAs were further explored using *post-hoc* comparisons with Bonferroni correction to control the familywise error rate at α = 0.05 (Armstrong, 2014).

To explore potential relationships between key variables, Pearson's correlation coefficients were calculated. This analysis examined associations among moral reasoning ability scores (DIT-2 N2), average HR during the conditions, moral decision-making accuracy (in VR), moral decision-making speed (in VR), and self-reported post-scenario stress levels (STAI-S). These correlations aimed to provide insight into the interplay between moral reasoning, stress responses, and decision-making under pressure (Cohen, 1988). Data management included handling outliers and missing data. Outliers in HR data, identified as values exceeding *z* = ± 3.29, were managed using winsorization (replacing outliers with the nearest non-outlier value) to maintain data integrity (Tabachnick and Fidell, 2013). Missing data, expected to be minimal (< 5%), were handled using listwise deletion for relevant analyses (Little and Rubin, 2019). Finally, a *post-hoc* power analysis was conducted using G^*^Power (Faul et al., 2007) to evaluate the achieved statistical power based on observed effect sizes for all non-significant main effects and interactions. This assessed the study's sensitivity to detect significant effects with the obtained sample size.

## 4 Results

### 4.1 Participant characteristics

A total of 120 adolescents (62 females, 58 males) aged 14-17 years (*M* = 15.8 years, *SD* = 0.72) participated in the study. Data from two participants were excluded due to technical difficulties with the VR scenario recording, resulting in a final sample size of 118 participants (60 females, 58 males). Participants were categorized into high (*n* = 59) and low (*n* = 59) moral reasoning ability groups based on a median split of their N2-scores on the Defining Issues Test-2 (DIT-2). The high moral reasoning ability group had a mean N2-score of 60.5 (SD = 8.2), while the low moral reasoning ability group had a mean N2-score of 40.3 (SD = 7.5). There were no significant differences between the high and low moral reasoning ability groups in terms of age, gender, or educational background (all *p* >.05).

### 4.2 Mixed-factors ANOVAs

#### 4.2.1 Heart rate reactivity

To investigate the effects of moral reasoning ability and the VR scenario on average heart rate (HR) during task engagement, a 2 (Moral Reasoning Ability Group: High vs. Low) x 2 (Condition: VR Scenario vs. Control) mixed-factors ANOVA was conducted. This analysis aimed to determine whether adolescents with higher moral reasoning ability displayed different physiological stress responses when exposed to social-moral conflict in a VR environment, as reflected by their average HR during the task compared to a baseline.

The analysis revealed a significant main effect of condition (F _(1, 116)_ = 23.78, *p* < 0.001, η^2^ = 0.170), indicating that participants exhibited a higher average HR during the VR scenario (M = 82.4 bpm, SD = 7.8) compared to the control condition (M = 74.1 bpm, SD = 6.2). This result suggests that the VR scenario successfully induced a state of physiological arousal, as evidenced by the increased heart rate relative to the control condition and, implicitly, their established baseline. Descriptive statistics for average HR across conditions are presented in Table 2.

**Table 2:** Mean heart rate (bpm) and Standard Deviations (SD) by condition.

**Condition**	**Mean HR (bpm)**	**SD (bpm)**
VR scenario	82.4	7.8
Control	74.1	6.2

Further examination of the data showed that the interaction effect between moral reasoning ability and condition was not statistically significant (F _(1, 116)_ = 1.24, *p* = 0.268, η2 = 0.011). This indicates that the effect of the VR scenario on HR (i.e., the increase from baseline or difference between conditions) did not differ significantly between the high and low moral reasoning ability groups. Descriptive statistics for HR by moral reasoning ability group and condition are provided in Table 3.

**Table 3:** Descriptive statistics of key variables.

**Variable**	**Group**	**Condition**	**N**	**Mean**	**SD**	**Min**	**Max**
Age (years)	Overall	-	118	15.8	0.72	14	17
Gender	Female	-	60	-	-	-	-
	Male	-	58	-	-	-	-
Moral reasoning (DIT-2 N2-Score)	High MR	-	59	60.5	8.2	50	75
	Low MR	-	59	40.3	7.5	25	50
Heart rate (bpm)	High MR	VR scenario	59	80.9	7.2	65.2	95.0
	High MR	Control	59	74.3	6.4	62.7	85.0
	Low MR	VR scenario	59	83.9	8.4	66.0	98.1
	Low MR	Control	59	73.9	6.1	63.0	85.3
Decision-making accuracy (%)	High MR	VR scenario	59	84.2	6.8	68.4	95.7
	High MR	Control	59	84.5	6.5	70.1	96.2
	Low MR	VR scenario	59	83.7	7.1	65.2	94.8
	Low MR	Control	59	83.9	7.3	67.8	95.1
Decision-making speed (seconds)	High MR	VR scenario	59	4.1	1.0	2.8	6.5
	High MR	Control	59	3.8	0.9	2.5	5.5
	Low MR	VR scenario	59	4.3	1.2	2.9	6.7
	Low MR	Control	59	4.0	1.1	2.6	5.8
Self-reported stress (STAI-S score)	High MR	VR scenario	59	41.5	7.0	28.1	58.0
	High MR	Control	59	38.5	6.0	25.7	50.0
	Low MR	VR Scenario	59	42.7	7.8	29.0	59.2
	Low MR	Control	59	39.3	6.4	26.0	51.4

MR, Moral Reasoning.

#### 4.2.2 Decision-making and self-reported stress

The second 2 (Moral Reasoning Ability Group: High vs. Low) x 2 (Condition: VR Scenario vs. Control) mixed-factors ANOVA examined how moral reasoning ability and the VR scenario condition influenced moral decision-making accuracy, moral decision-making speed, and self-reported stress (STAI-S). It is important to note that moral decision-making accuracy and speed were only assessed during the VR scenario, as the control condition (book sorting) did not involve comparable moral dilemmas or decision points relevant to these specific measures. Therefore, while the ANOVA structure includes 'Condition,' the interpretation for these specific dependent variables for the control condition reflects the time taken and “accuracy” of completing the neutral sorting task, rather than moral judgment.

### 4.3 Decision-making accuracy

The analysis of moral decision-making accuracy in the VR scenario revealed no significant main effects of moral reasoning ability (F _(1, 116)_ = 0.78, *p* = 0.378, η^2^ = 0.007) or condition (F _(1, 116)_ = 1.20, *p* = 0.274, η^2^ = 0.010), nor a significant interaction effect between moral reasoning ability and condition (F _(1, 116)_ = 0.02, *p* = 0.883, η2 < 0.001). These results suggest that both high and low moral reasoning ability participants performed similarly in terms of making accurate choices about how to facilitate the discussion within the VR scenario. The descriptive statistics for decision-making accuracy are presented in Table 4.

**Table 4:** Mean decision-making accuracy (percentage) and Standard Deviations (SD) by condition and moral reasoning ability group.

**Condition**	**Moral reasoning ability group**	***N***	**Mean accuracy (%)**	**SD(%)**
VR Scenario	High	59	84.2	6.8
	Low	59	83.7	7.1
Control	High	59	84.5	6.5
		Low	59	83.9	7.3

The lack of significant differences in moral decision-making accuracy indicates that the VR scenario's social-moral conflict did not differentially impact participants based on their level of moral reasoning ability. Both high and low moral reasoning ability groups were able to navigate the moral dilemmas presented in the VR scenario with similar accuracy. For the control condition, the accuracy reflects adherence to sorting rules, which remained consistently high across groups, as expected from a straightforward cognitive task.

### 4.4 Decision-making speed

The analysis revealed a significant main effect of condition (F _(1, 116_) = 4.21, *p* = 0.042, η^2^ =.036) on decision-making speed. Participants took slightly longer to make decisions within the VR scenario (M = 4.2 seconds, SD = 1.1) compared to the control condition (M = 3.9 seconds, SD = 1.0). This difference suggests that the increased cognitive demands of navigating the VR environment and processing social cues during decision-making may have slowed participants down. The descriptive statistics for decision-making speed are presented in Table 5.

**Table 5:** Mean decision-making speed (seconds) and Standard Deviations (SD) by condition.

**Condition**	**Mean speed(seconds)**	**SD(seconds)**
VR scenario	4.2	1.1
Control	3.9	1.0

There were no significant main effects of moral reasoning ability (F _(1, 116)_ = 1.32, *p* = 0.252, η^2^ =.012) or interaction effects between moral reasoning ability and condition (F (_1, 116_) = 0.87, *p* = 0.352, η^2^ = 0.008) on decision-making speed. This indicates that both high and low moral reasoning ability participants made decisions at a similar pace within the VR scenario, suggesting that moral reasoning ability did not influence the speed of moral decision-making under social pressure. For the control condition, this speed reflects the rate of completing the organizational task.

### 4.5 Self-reported stress

The analysis of self-reported stress using the STAI-S scores revealed a significant main effect of condition (F _(1, 116)_ = 18.32, *p* < 0.001, η^2^ = 0.139). Participants reported higher levels of stress (*M* = 42.1, *SD* = 7.4) after experiencing the VR scenario compared to the control condition (*M* = 38.9, *SD* = 6.2). This finding supports the notion that the VR scenario successfully induced a state of perceived stress. The descriptive statistics for self-reported stress (STAI-S) are presented in Table 6.

**Table 6:** Mean Self-Reported Stress Scores (STAI-S) and Standard Deviations (SD) by condition.

**Condition**	**Mean STAI-S score**	**SD**
VR Scenario	42.1	7.4
Control	38.9	6.2

The main effect of moral reasoning ability (F _(1, 116)_ = 0.08, *p* = 0.778, η^2^ < 0.001) and the interaction effect between moral reasoning ability and condition (F _(1, 116_) = 0.01, *p* = 0.907, η^2^ < 0.001) on self-reported stress (STAI-S) were not statistically significant. This suggests that adolescents with high or low moral reasoning ability reported similar levels of stress after experiencing the VR scenario.

A *post-hoc* power analysis was conducted for all non-significant findings. For the non-significant interaction effect of moral reasoning ability and condition on HR reactivity (observed η^2^ = 0.011), the achieved power was.21. For the non-significant main effect of moral reasoning ability on decision-making accuracy (observed η^2^ = 0.007), the achieved power was.14. For the non-significant interaction effect on decision-making accuracy (observed η^2^ < 0.001), power was < 0.05. Similarly, for the non-significant main effect of moral reasoning ability on decision-making speed (observed η^2^ = 0.012), achieved power was 0.23, and for its interaction effect (observed η^2^ = 0.008), power was 0.15. Finally, for the non-significant main effect of moral reasoning ability on self-reported stress (observed η^2^ < 0.001), achieved power was < 0.05, and for its interaction effect (observed η^2^ < 0.001), power was also < 0.05. These low achieved power values indicate that the study may have been underpowered to detect small to medium effects for these particular variables.

Overall, these results indicate that while the VR scenario effectively induced physiological and perceived stress, the level of moral reasoning ability did not significantly modulate decision-making accuracy, decision-making speed, or self-reported stress in this study. These findings suggest that both high and low moral reasoning ability adolescents responded similarly to social-moral conflict in terms of their decision-making performance and stress levels. Future research could explore additional factors that might interact with moral intelligence to influence these outcomes, providing a more nuanced understanding of the mechanisms underlying stress and decision-making in morally challenging situations.

## 5 Discussion

This study investigated the interplay between the moral reasoning component of Moral Intelligence (MI), social-moral conflict, and stress regulation in adolescents using an immersive VR simulation. The findings contribute to our understanding by revealing complex dynamics and offering insights into the contextual and developmental moderators of moral functioning under pressure. A key finding is that, despite the effectiveness of the VR scenario in inducing significant physiological arousal (increased heart rate) and subjective state anxiety, higher moral reasoning ability (operationalized via DIT-2 N2 scores) did not significantly buffer adolescents against these stress responses, nor did it lead to significantly better decision-making accuracy or faster decisions within this challenging context. This outcome, while contrasting with some theoretical expectations regarding MI's protective role, provides crucial insights into the boundary conditions under which moral reasoning influences behavior and stress regulation, particularly highlighting the potent impact of high-pressure social situations and adolescent developmental factors.

Focusing first on physiological responses, the significant increase in average heart rate (HR) during the VR scenario compared to the control condition confirms the effectiveness of VR as a tool for inducing physiological stress. This aligns with previous research indicating that immersive VR environments can replicate real-life stressors and elicit genuine physiological responses (Ni et al., 2023). The capability of VR to create realistic, stress-inducing environments is well-documented and increasingly utilized in psychological research due to high ecological validity and tight experimental control (Parsons and Rizzo, 2008). Despite the successful stress induction, the lack of a significant interaction effect between moral reasoning ability and condition suggests that higher moral reasoning ability did not significantly buffer the physiological stress response as hypothesized. This finding contrasts with some prior studies associating high MI with better stress management (Asefi Far et al., 2023; Lennick and Kiel, 2011), such as findings that higher moral reasoning correlated with lower anxiety (Asefi Far et al., 2023) or suggestions that MI provides necessary cognitive and emotional tools for stress regulation (Lennick and Kiel, 2011).

A compelling explanation for this result is that the intensity of the simulation may have overwhelmed the cognitive resources required to deploy higher-order reasoning skills. This is especially pertinent for adolescents, a developmental period characterized by still-maturing capacities for emotional and cognitive regulation (Steinberg, 2005). The acute demands of the task, combined with heightened adolescent emotional reactivity, likely constrained participants' ability to access and apply their abstract reasoning abilities effectively. In essence, while adolescents may possess principled moral reasoning (Kohlberg, 1984), the neurobiological effects of acute stress can temporarily disrupt the very executive functions needed to implement it (Lupien et al., 2007). This provides a developmental account for why moral reasoning did not act as a protective factor in this demanding scenario.

Beyond developmental factors, the collectivistic cultural context of the Chinese adolescent participants offers a critical interpretive lens. In a culture that highly values social harmony and relational obligations (Markus and Kitayama, 1991), the pressure to maintain interpersonal peace can supersede abstract, internal moral principles. This cultural script may have reframed the primary task for participants from discerning the most “principled” solution to managing an immediate social conflict to restore harmony. Furthermore, cultural values that encourage suppressing strong emotions to preserve group cohesion likely promote learned relational coping responses over individual strategies derived from cognitive reasoning. This emphasis on relational, rather than self-regulation, provides a compelling cultural explanation for the uniform stress responses across groups.

This interpretation aligns with the distinction between “cold” and “hot” cognitive processes. Moral reasoning is a “cold” cognitive faculty, concerned with abstract principles. The VR scenario, however, created a “hot” context, demanding immediate emotional regulation and social processing. In such conditions, emotional regulation may act as a prerequisite, creating the necessary mental space for an adolescent to apply their moral reasoning skills. An adolescent might *know* the principled way to resolve a conflict but lack the composure to implement that knowledge when faced with social threat (Mikolajczak et al., 2009). Without well-developed emotional intelligence, the cognitive resources for complex moral reasoning may simply be inaccessible under duress.

Similarly, empathy—another core component of MI—likely plays a crucial role not captured by our focus on reasoning alone. Cognitive empathy, or perspective-taking, might have enabled participants to better understand the virtual classmates' arguments, potentially reducing the perceived conflict and mitigating the stress response (Davis, 1983). This suggests that MI's protective effects in such scenarios arise not from a single component in isolation, but from a synergistic interplay between them: empathy to understand the social context, emotional intelligence to manage the stress, and moral reasoning to guide the ultimate decision. Therefore, our null finding for moral reasoning does not diminish the potential importance of MI; instead, it illuminates its multifaceted nature and highlights the interdependence of its components in navigating complex social-moral challenges.

Turning to cognitive performance, the analyses of decision-making accuracy and speed also revealed no significant main or interaction effects involving moral reasoning ability. This indicates that moral reasoning ability did not significantly influence the ability to make accurate choices or the time taken to make decisions within the simulated moral dilemmas presented in the VR scenario. This outcome diverges from previous studies generally associating higher MI with more ethical or prosocial decision-making (Small and Lew, 2021; Tanner and Christen, 2014). The high-stress context imposed by the VR task in this study, however, may have diminished these expected effects.

Crucially, the divergence of our findings from prior literature may be a direct consequence of our methodology. Much of the existing research relies on static, text-based moral dilemmas that assess abstract, “offline” judgment in low-pressure contexts. Our study, by contrast, used an immersive VR simulation with dynamic, situationally arising interactions with virtual avatars. This method provides a more ecologically valid test of moral decision-making by forcing participants to manage not just a cognitive puzzle, but also real-time social and emotional pressures. It is therefore plausible that the documented benefits of high moral reasoning are most apparent in abstract scenarios, but that these advantages attenuate significantly when an adolescent is faced with the “hot,” immediate, and socially demanding nature of a life-like conflict. In this light, our null finding is not necessarily a contradiction of past work, but rather a clarification of its boundary conditions, suggesting the link between abstract reasoning and in-the-moment behavior is more fragile than previously understood.

Increased cognitive demands and social pressures inherent in the scenario could have created conditions where the typical advantages of high moral reasoning ability were less operative, potentially because stress can impair cognitive functioning and overload executive functions (Lupien et al., 2007). Interestingly, participants in the VR scenario took slightly longer to make decisions compared to the control condition. This finding contrasts with some suggestions that stress invariably speeds up decision-making (e.g., Huy and Phuc, 2024) but aligns with research indicating that stress can slow decision processes during complex, morally relevant tasks that may induce conflict and require careful consideration (Starcke and Brand, 2012). It is plausible that the complexity and social-moral nature of the VR scenario prompted more cautious, deliberative processing across participants, reflected in the increased response times, rather than faster, reactive choices (Kahneman, 2011). The absence of significant moral reasoning ability effects on decision-making speed further underscores the potential limits of moral reasoning under high-pressure conditions. It appears the acute stress and complexity may have largely driven decision timing, potentially overwhelming cognitive frameworks provided by moral reasoning ability and leading to more uniformly cautious decision-making. This highlights how situational factors can moderate the influence of moral reasoning, necessitating further exploration across different contexts (Buon et al., 2016; Rest et al., 1999).

Finally, regarding subjective experience, the higher self-reported state anxiety levels following the VR scenario compared to the control condition confirm the psychological impact of the induced social-moral conflict, consistent with evidence that VR effectively elicits emotional responses (Baumgartner et al., 2008). The immersive and challenging design likely contributed to these reports. Yet, similar to the physiological and cognitive findings, the level of moral reasoning ability did not significantly affect these self-reported state anxiety scores. Adolescents with higher moral reasoning ability perceived the VR-induced stress similarly to those with lower moral reasoning ability, which contrasts with studies linking higher MI or related constructs like emotional intelligence to lower perceived stress and better emotional regulation (Downey et al., 2010; Pérez-Fuentes et al., 2019). Plausible explanations mirror those for the physiological findings: the intense, immediate nature of the VR conflict may have overwhelmed typical coping strategies associated with moral reasoning, especially given the developmental stage of the participants. Adolescents possess heightened emotional reactivity and less mature cognitive control compared to adults (Casey et al., 2008), potentially constraining their ability to leverage moral reasoning effectively for emotional regulation in this demanding setting (Smetana, 2011). Context-dependent effects, where the relationship between MI and stress varies across situations and populations (Irfan and Kausar, 2018), may also explain why moral reasoning did not buffer subjective stress in this specific, challenging VR environment.

## 6 Implications

This study examined how moral reasoning ability, as a component of moral intelligence (MI), interacts with stress and decision-making during simulated social-moral conflict in adolescents. The VR scenario effectively induced stress, yet higher moral reasoning ability, as assessed by the DIT-2, did not buffer physiological or subjective stress responses, nor did it significantly improve decision-making accuracy or speed under pressure. These results highlight the significant role of situational and developmental context in shaping the effectiveness of moral reasoning during adolescence.

The findings offer several theoretical implications for understanding moral reasoning's role within MI, stress regulation, and decision-making in adolescents. Firstly, they underscore the importance of situational and developmental context in evaluating the role of moral reasoning. While higher MI, often encompassing robust moral reasoning, is generally linked to better stress management, its effectiveness appears reduced in high-pressure, socially complex settings like the VR scenario, particularly for adolescents whose abstract reasoning is still emerging and can be easily disrupted by stress (Kohlberg, 1984; Lupien et al., 2007). This aligns with Kohlberg's developmental stages, suggesting that adolescents' transition to principled reasoning may falter under intense stress, limiting the protective effects that might otherwise be associated with advanced moral reasoning.

Secondly, the study highlights the need to integrate MI's multiple dimensions—moral reasoning, empathy, and moral identity—when assessing its full impact. The null findings here, specific to moral reasoning, suggest these components interact complexly, possibly requiring the concurrent development of emotional intelligence or resilience to fully mitigate stress (Holian, 2006; Alhadabi et al., 2019). Future research should explore these interactions further, investigating how the interplay of these various facets of MI contributes to coping with social-moral dilemmas.

From a practical standpoint, the null findings—that high moral reasoning ability did not buffer stress or enhance decision-making in this specific high-pressure context—suggest that relying solely on fostering moral reasoning may not suffice in acutely demanding situations. Interventions should therefore focus not only on developing moral reasoning but also on applied skills like general stress management and decision-making under pressure, potentially using VR as an effective training tool (Ni et al., 2023). Supportive environments, such as peer support or mentorship programs, are also critical to reinforce ethical behavior and help adolescents navigate moral dilemmas when cognitive moral reasoning alone falls short (Smetana, 2011; Eisenberg et al., 2006).

## 7 Limitations and future directions

While providing valuable insights, this study has several limitations that suggest directions for future research. First, findings may not generalize beyond the specific context of the Beijing high schools sampled via convenience methods. Cultural and educational factors unique to this setting could influence the development and expression of moral reasoning ability and other MI components. Future research replicating this design across diverse geographical, cultural, and socioeconomic adolescent populations is needed to establish external validity and understand potential contextual variations in moral functioning (Henrich et al., 2010).

Second, relying solely on the DIT-2 limits the assessment of MI to the moral reasoning component, potentially overlooking other crucial dimensions like empathy and moral identity. To gain a more comprehensive view of MI's influence, future studies should employ multi-dimensional assessments, incorporating measures such as the Moral Identity Scale (Aquino and Reed, 2002) and the Interpersonal Reactivity Index (Davis, 1983), possibly alongside behavioral or physiological indicators, to better understand how various MI facets influence stress and decision-making.

Third, the VR scenario, while stress-inducing, inevitably simplifies the complexity and unpredictability of real-world conflicts. Future work could benefit from exploring more varied or dynamic VR scenarios that allow for a broader range of moral choices and social interactions. Incorporating longitudinal designs would also be valuable for tracking the development of moral reasoning ability and its influence on responses to moral challenges over time, complemented perhaps by ecological momentary assessment (EMA) to capture real-world navigation of dilemmas (Shiffman et al., 2008).

Fourth, potential VR novelty effects, where unfamiliarity with the technology causes distraction or discomfort, might have influenced responses. Although a brief familiarization was included, future studies could employ longer or more extensive acclimatization periods to minimize such effects and ensure more natural engagement with the VR tasks.

Finally, this study did not measure or control for potentially relevant individual differences like baseline stress levels, coping styles, or prior experience with moral conflict. Future research should assess such variables to examine their influence either as covariates or potential moderators (alongside factors like personality, social support, or moral education exposure) of the effectiveness of moral reasoning ability in stressful situations.

## 8 Conclusion

In conclusion, this study demonstrates that for adolescents, the capacity for high-level moral reasoning may be insufficient to buffer against the stress of an immediate social-moral conflict or to improve decision-making within it. The findings suggest that the practical application of moral principles under pressure is heavily moderated by situational intensity and developmental factors.

Beyond these theoretical insights, this study makes a significant methodological contribution to the field of moral psychology. By using immersive VR, we were able to move beyond the traditional, vignette-based assessments that have long dominated moral reasoning research. Such methods, while valuable, primarily capture abstract, “offline” moral judgment and struggle to simulate the acute stress and social pressures of real-world dilemmas. Our VR paradigm offers a crucial bridge between abstract cognition and in-the-moment behavior, creating an ecologically valid environment that elicits genuine physiological and emotional stress responses. This approach, which uniquely combines tight experimental control with realistic social simulation, opens new avenues for investigating the complex interplay of cognition, emotion, and behavior in moral contexts. It represents a critical step forward in understanding what it takes not only to know what is right, but to act on that knowledge when it matters most.

## Data Availability

The data analyzed in this study is subject to the following licenses/restrictions: Datasets generated and analyzed in this study are available from the corresponding author upon reasonable request. Requests to access these datasets should be directed to Jianbao Zhang, zhangjianbaobwu@outlook.com.

## References

[B1] Alhadabi A. Aldhafri S. Alkharusi H. Al-Harthy I. Alrajhi M. AlBarashdi H. (2019). Modelling parenting styles, moral intelligence, academic self-efficacy and learning motivation among adolescents in grades 7–11. Asia Pacific J. Educ. 39, 133–153. 10.1080/02188791.2019.1575795

[B2] Aquino K. Reed A. (2002). The self-importance of moral identity. J. Pers. Soc. Psychol. 83, 1423–1440. 10.1037/0022-3514.83.6.1423 12500822

[B3] Armstrong R. A. (2014). When to use the Bonferroni correction. Ophthal. Physiol. Optics 34, 502–508. 10.1111/opo.12131 24697967

[B4] Asefi Far F. Ayoobi E. Jafari K. Salmabadi M. (2023). The relationship between moral intelligence and corona anxiety in students:t mediating role of distress tolerance. Iran. J. Rehabil. Res. 9, 9–18.

[B5] Awad N. H. A. Ashour H. M. A. A. (2020). The relationship between emotional intelligence, moral distress and work engagement as perceived by critical care nurses. Int.J. Adv. Nurs. Manag. 8, 237–248. 10.5958/2454-2652.2020.00060.8

[B6] Bajovic M. Rizzo K. (2021). Meta-moral cognition: bridging the gap among adolescents' moral thinking, moral emotions and moral actions. Int. J. Adolesc. Youth 26, 1–11. 10.1080/02673843.2020.1867206

[B7] Bandura A. (1999). Moral disengagement in the perpetration of inhumanities. Pers. Soc. Psychol. Rev. 3, 193–209. 10.1207/s15327957pspr0303_3 15661671

[B8] Barida M. Prasetiawan H. Muarifah A. (2019). The development of self-managementtechnique for improving students' moral intelligence. Int. J. Educ. Res. Rev. 4, 660–669. 10.24331/ijere.628483

[B9] Baumgartner T. Valko L. Esslen M. Jäncke L. (2008). Neural correlate of spatial presence in an arousing and noninteractive virtual reality: an EEG and psychophysiology study. CyberPsychol. Behav. 11, 16–21. 10.1089/cpb.2006.9.30 16497116

[B10] Berntson G. G. Quigley K. S. Lozano D. (2007). “Cardiovascular psychophysiology,” in Handbook of psychophysiology, 3rd Edn.eds. J. T. Cacioppo, L. G. Tassinary, and G. G. Berntson (Cambridge University Press), 182–210. 10.1017/CBO9780511546396.008

[B11] Blasi A. (1980). Bridging moral cognition and moral action: a critical review of the literature. Psychol. Bull. 88, 1–45. 10.1037/0033-2909.88.1.1

[B12] Boegershausen J. Aquino K. Reed I. I. A. (2015). Moral identity. Curr. Opin. Psychol. 6, 162–166. 10.1016/j.copsyc.2015.07.017

[B13] Bucciarelli M. Khemlani S. Johnson-Laird P. N. (2008). The psychology of moral reasoning. Judg. Dec. Making 3, 121–139. 10.1017/S1930297500001479

[B14] Buon M. Habib M. Frey D. (2016). Moral development: Conflicts and compromises. In J. A. Sommerville and J. Decety (Eds.), Social Cognition (p. 147–168). Routledge. 10.31234/osf.io/nznt6_v1

[B15] Casey B. J. Getz S. Galvan A. (2008). The adolescent brain. Dev. Rev. 28, 62–77. 10.1016/j.dr.2007.08.003 18688292 PMC2500212

[B16] Chida Y. Hamer M. (2008). Chronic psychosocial factors and acute physiological responses to laboratory-induced stress in healthy populations: a quantitative review of 30 years of research. Psychol. Bull. 134, 829–856. 10.1037/a0013342 18954159

[B17] Chorus C. G. (2015). Models of moral decision making: Literature review and research agenda for discrete choice analysis. J. Choice Modell. 16, 69–85. 10.1016/j.jocm.2015.08.001

[B18] Chowdhury R. M. (2017). Emotional intelligence and consumer ethics: the mediating role of personal moral philosophies. J. Bus. Ethics 142, 527–548. 10.1007/s10551-015-2733-y

[B19] Cohen J. (1988). Statistical Power Analysis for the Behavioral Sciences, 2nd Edn. Lawrence Erlbaum Associates.

[B20] Cooley S. Elenbaas L. Killen M. (2012). Moral judgments and emotions: adolescents' evaluations in intergroup social exclusion contexts. New Dir. Youth Dev. 2012, 41–57. 10.1002/yd.20037 23359443 PMC4096118

[B21] Davis M. H. (1983). Measuring individual differences in empathy: evidence for a multidimensional approach. J. Pers. Soc. Psychol. 44, 113–126. 10.1037/0022-3514.44.1.113

[B22] Downey L. A. Johnston P. J. Hansen K. Birney J. Stough C. (2010). Investigating the mediating effects of emotional intelligence and coping on problem behaviours in adolescents. Aus. J. Psychol. 62, 20–29. 10.1080/00049530903312873

[B23] Eisenberg N. Spinrad T. L. Sadovsky A. (2006). “Empathy-related responding in children,” in Handbook of Moral Development, eds. M. Killen and J. G. Smetana (Psychology Press), 517-549.

[B24] Faul F. Erdfelder E. Lang A. G. Buchner A. (2007). G^*^Power 3: a flexible statistical power analysis program for the social, behavioral, and biomedical sciences. Behav. Res. Methods 39, 175–191. 10.3758/BF03193146 17695343

[B25] Field A. (2018). Discovering Statistics Using Ibm Spss Statistics 5th Edn. SAGE Publications.

[B26] Fourie C. (2015). Moral distress and moral conflict in clinical ethics. Bioethics 29, 91–97. 10.1111/bioe.12064 24602097

[B27] Gibbs J. C. Basinger K. S. Grime R. L. Snarey J. R. (2007). Moral judgment development across cultures: revisiting Kohlberg's universality claims. Dev. Rev. 27, 443–500. 10.1016/j.dr.2007.04.001

[B28] Hannah S. T. Thompson R. L. Herbst K. C. (2020). Moral identity complexity: situated morality within and across work and social roles. J. Manag. 46, 726–757. 10.1177/0149206318814166

[B29] Hardy S. A. Carlo G. (2005). Identity as a source of moral motivation. Hum. Dev. 48, 232–256. 10.1159/000086859

[B30] Henrich J. Heine S. J. Norenzayan A. (2010). The weirdest people in the world? Behav. Brain Sci. 33, 61–83. 10.1017/S0140525X0999152X 20550733

[B31] Holian R. (2006). Management decision making, ethical issues and “emotional” intelligence. Manag. Dec. 44, 1122–1138. 10.1108/00251740610690658

[B32] Huy P. Q. Phuc V. K. (2024). Sustainable decision making in the time of uncertainty: does moral intelligence make it different? Pacific Asia J. Assoc. Inform. Syst. 16:8. 10.2478/fman-2024-0005

[B33] Irfan S. Kausar R. (2018). Emotional intelligence as predictor of moral judgment in adolescents. J. Res. Ref. Educ. 12, 204–228.

[B34] Kahneman D. (2011). Thinking, Fast and Slow. Farrar, Straus and Giroux.

[B35] Kohlberg L. (1984). Essays on Moral Development: Vol. 2. The Psychology of Moral Development. Harper and Row.

[B36] Kornilova T. Chigrinova I. (2014). Personal values, moral development, and emotional intelligence in the regulation of choice in situations that involve interpersonal interactions. Psychol. J. High. Sch. Econ. 11, 56–74.

[B37] Krol S. A. Bartz J. A. (2022). The self and empathy: lacking a clear and stable sense of self undermines empathy and helping behavior. Emotion 22:1554. 10.1037/emo0000943 33570970

[B38] Lea R. Davis S. K. Mahoney B. Qualter P. (2023). Do emotionally intelligent adolescents flourish or flounder under pressure? Linking emotional intelligence to stress regulation mechanisms. Pers. Individ. Diff. 201:111943. 10.1016/j.paid.2022.111943

[B39] Lehalle H. (2020). “Moral development in adolescence: how to integrate personal and social values,”? in Handbook of Adolescent Development (Psychology Press), 118-134. 10.4324/9780203969861-7

[B40] Lennick D. Kiel F. (2011). Moral Intelligence 2.0: Enhancing Business Performance and Leadership Success in Turbulent Times. Pearson Prentice Hall.

[B41] Little R. J. A. Rubin D. B. (2019). Statistical Analysis With Missing Data, 3rd Edn. John Wiley and Sons. 10.1002/9781119482260

[B42] Lupien S. J. Maheu F. Tu M. Fiocco A. Schramek T. E. (2007). The effects of stress and stress hormones on human cognition: Implications for the field of brain and cognition. Brain Cogn. 65, 209–237. 10.1016/j.bandc.2007.02.007 17466428

[B43] Markus H. R. Kitayama S. (1991). Culture and the self: implications for cognition, emotion, and motivation. Psychol. Rev. 98, 224–253. 10.1037/0033-295X.98.2.224

[B44] Mikolajczak M. Petrides K. V. Hurry J. (2009). Adolescents choosing self-harm as an emotion regulation strategy: the protective role of trait emotional intelligence. Br. J. Clin. Psychol. 48, 181–193. 10.1348/014466508X386027 19054434

[B45] Narvaez D. (2010). The emotional foundations of high moral intelligence. New Dir. Child Adolesc. Dev. 2010, 77–94. 10.1002/cd.276 20872605

[B46] Ni B. K. Burns B. D. Mak K. K. Lah S. Silva D. S. Goldwater M. B.. (2023). To kill or not to kill: a systematic literature review of high-stakes moral decision-making measures and their psychometric properties. Front. Psychol. 13:1063607. 10.3389/fpsyg.2022.1063607 36698597 PMC9869153

[B47] Parsons T. D. Rizzo A. A. (2008). Affective outcomes of virtual reality exposure therapy for anxiety and specific phobias: a meta-analysis. J. Behav. Ther. Exp. Psychiatr. 39, 250–261. 10.1016/j.jbtep.2007.07.007 17720136

[B48] Pérez-Fuentes M. D. C. Molero Jurado M. D. M. Barragán Martín A. B. Gazquez Linares J. J. (2019). Family functioning, emotional intelligence, and values: analysis of the relationship with aggressive behavior in adolescents. Int. J. Environ. Res. Public Health 16:478. 10.3390/ijerph16030478 30736326 PMC6388189

[B49] Rest J. R. Narvaez D. Bebeau M. J. Thoma S. J. (1999). Postconventional Moral Thinking: A Neo-Kohlbergian Approach. Lawrence Erlbaum Associates. 10.4324/9781410603913

[B50] Shiffman S. Stone A. A. Hufford M. R. (2008). Ecological momentary assessment. Annu. Rev. Clin. Psychol. 4, 1–32. 10.1146/annurev.clinpsy.3.022806.091415 18509902

[B51] Small C. Lew C. (2021). Mindfulness, moral reasoning and responsibility: towards virtue in ethical decision-making. J. Bus. Ethics 169, 103–117. 10.1007/s10551-019-04272-y

[B52] Smetana J. G. (2011). “Adolescents' social reasoning and relationships with parents: conflicts and coordinations within and across domains,” in Adolescent Vulnerabilities and Opportunities: Constructivist and Developmental Perspectives, eds. M. J. Bunge and P. L. Ganter (Cambridge University Press), 139-158. 10.1017/CBO9781139042819.009

[B53] Snarey J. R. (1985). Cross-cultural universality of social-moral development: a critical review of Kohlbergian research. Psychol. Bull. 97:202. 10.1037/0033-2909.97.2.202 3983300

[B54] Snarey J. R. Reimer J. Kohlberg L. (1985). Development of social-moral reasoning among Kibbutz adolescents: a longitudinal cross-cultural study. Dev. Psychol. 21, 3–17. 10.1037/0012-1649.21.1.3

[B55] Spielberger C. D. Gonzalez-Reigosa F. Martinez-Urrutia A. Natalicio L. F. Natalicio D. S. (1971). The state-trait anxiety inventory. Rev. Int. Psicol./Interam. J. Psychol. 5, 145–158.

[B56] Starcke K. Brand M. (2012). Decision making under stress: a selective review. Neurosci. Biobehav. Rev. 36, 1228–1248. 10.1016/j.neubiorev.2012.02.003 22342781

[B57] Steinberg L. (2005). Cognitive and affective development in adolescence. Trends Cogn. Sci. 9, 69–74. 10.1016/j.tics.2004.12.005 15668099

[B58] Stevens J. P. (2012). Applied Multivariate Statistics for the Social Sciences, 5th Edn. Routledge. 10.4324/9780203843130

[B59] Tabachnick B. G. Fidell L. S. (2013). Using Multivariate Statistics, 6th Edn. Pearson.

[B60] Tanner C. Christen M. (2014). “Moral intelligence – a framework for understanding moral competences,” in Empirically Informed Ethics: Morality Between Facts and Norms, eds. M. Christen, C. van Schaik, J. Fischer, M. Huppenbauer, and C. Tanner (Springer), 119-136. 10.1007/978-3-319-01369-5_7

[B61] Turiel E. (2002). The Culture of Morality: Social Development, Context, and Conflict. Cambridge University Press. 10.1017/CBO9780511613500

[B62] Wallace J. D. (2019). Moral Relevance and Moral Conflict. Cornell University Press.

[B63] Weber J. (2019). Understanding the millennials' integrated ethical decision-making process: assessing the relationship between personal values and cognitive moral reasoning. Bus. Soc. 58, 1671–1706. 10.1177/0007650317726985

[B64] White J. Bandura A. Bero L. A. (2009). Moral disengagement in the corporate world. Account. Res. 16, 41–74. 10.1080/08989620802689847 19247852

[B65] Zhang Q. Thoma S. J. (2016). An empirical cross-cultural study of moral judgment development in mainland China. Ethics Prog. 7, 228–245. 10.14746/eip.2016.1.15

